# Domestic Summary

**Published:** 1838-06-06

**Authors:** 


### DOMESTIC SUMMARY.

Professor Dunglison was elected one of the Physicians to the Philadelphia (Alms-House) Hospital, on the 21st of May, in the room of Dr. Stewardson, who resigned the situation upon being appointed to the Pennsylvania Hospital.

*Medical Convention.*—The April number of the Western Journal of the Medical and Physical Sciences calls particular attention to a series of resolutions, on the subject of medical education, passed by the Medical Convention of Ohio, during its session at Columbus, in January, 1838. One of these resolutions suggests to the various schools of the Union the expediency of sending delegates to a Convention, in some central city, that might devise correctives for many of the imperfections which now exist in their organization and administration. The Western Journal proposes Philadelphia as the proper place for such a meeting, the University of Pennsylvania to appoint the time and give the invitation.

*Medical College of Georgia.*—Some important changes have been lately made in the organization of this Institution. The number of professorships is limited to seven, and the length of the course of lectures reduced to that which is adopted in other medical schools, commencing on the second Monday of November, annually. An appropriate address was delivered to the graduates of the college, on the second of April, by the Rev. Elijah Sinclair, one of the board of trustees.

*Report of several cases of successful operation for club-foot by the division of the Tendo-Achillis, with remarks.* By W. Detmold, M. D., of New York.

Dr. Detmold, in the May number of the American Journal of the Medical Sciences, reports three cases of club-feet in children in which the deformity was entirely removed by division of the tendo-Achillis, and subsequent extension. The operation was first performed by Lorens, of Frankford in 1784, afterwards by Michaelis, in 1796, and again by Delpech, in 1816, with what success is not accurately ascertained. The operation had sunk into oblivion, when it was revived in 1830, by Dr. Louis Stromeyer, and so materially modified as to bestow on him valid claims to originality.

It may be well before describing the *modus operandi* to take a survey of the condition of the parts implicated. That the deformity depends upon abnormal muscular action, is evident from the possibility of cure in early childhood. The defect, of course, does not depend upon the bones. The disproportion in size and strength between the extending and flexing muscles is no where greater than in the leg.

"The extensors of the foot having overcome the contraction of the flexors, the heel is drawn up, and the weight of the whole body, instead of resting on the whole sole, is supported by a small portion only of this part; namely, the inferior extremity of the metatarsal bones, the toes being naturally kept in a constant state of almost complete extension; thus that species of club-foot is formed which is called *pes equinus*. Or, while the heel is drawn up, the point of the foot is at the same time drawn inwards, so that the weight of the body rests on the outer and upper part of the foot; this forms the highest degree of vaius, the real club-foot.

After this state of things has existed for some time, the flexors of the foot being entirely out of action, lose all their strength, the muscles of the calf being in a constant state of contraction, are drawn up, and become thinner, till at last the calf disappears entirely. The ligaments of the tarsus and metatarsus are stretched on one side and contracted on the other; that part of the foot which touches the ground being covered with a thick callus. If the point of the foot or of both feet is turned inwards, the patient, in order to get one foot round the other, is obliged to make a rotatory motion of the knee joint, which eventually affects and weakens the joint very much."

In infants, where the bones, ligaments and muscles are still in a plastic condition, suitable mechanical means may overcome the deformity. After a certain period, however, "any attempt to stretch the contracted muscles become so painful that the patient cannot bear it; besides, the extension soon begins to act as a stimulus upon the contracted muscles, and produce new and convulsive contractions, which frustrate every attempt at a cure, and increase the evil. This circumstance has suggested the idea of dividing the tendo-achillis, to counteract in this way the contractions of the gastrocnemii muscles. The operation, however, is here not the final aim; it merely produces a condition of parts favorable to the subsequent treatment; we allude to the cicatrix, which unites the extremities of the cut tendon; for the tendon is first divided, then the extremities are united again and healed, and after that the treatment of the disease begins."

It is a well established fact, that most of the tissues, when divided, heal by an intermediate substance, analagous to, but not identical with them. This intermediate substance in the cicatrix of tendons is peculiar, and possesses extensibility. Upon this fact the success of the operation is based.

Dr. Detmold now proceeds to lay down the following rules for performing the operation.

"The patient having been prepared for the operation, by being restrained from walking for a few days previously, and by daily bathing his feet, he is laid down flat on his face, an assistant presses the knee down upon the bed,—the surgeon with his left hand takes hold of the heel, and introduces a narrow, pointed, and curved bistoury, about one or two inches above the insertion of the tendo-achillis, between the bone and this tendon, the edge of the knife turned towards the latter, and pushes it through, so that the point comes out on the other side, without making the cutaneous wounds wider than the blade of the instrument. The surgeon then draws with his left hand the heel down as much as possible, and thus divides the tendon which separates with a jerk and a distinct noise. As soon as this is perceived, and not until then, the instrument is withdrawn in the same way in which it had been introduced. Precaution must be taken not to make the two cutaneous wounds larger than necessary, and not to get the point of the knife between the fibres of the tendon, but to go clearly round it, so as to divide it in its whole thickness. The wounds, which generally bleed very little, are covered with common court-plaster.

The next object is to bring the divided extremities of the tendon together, and we think the bandage best calculated to fulfil this, is a modification of Desault's bandage for the rupture of the tendo-achillis, with a bent splint over the knee, avoiding at the same time all pressure on the wound, as this is apt to produce convulsive contractions of the gastrocnemii muscles. This bandage leaves the wound free, and allows the use of cold fomentations, to keep down inflammation. After forty-eight hours the external wounds are generally healed, and the cohesion of the tendon is so far advanced as to admit the application of the extending apparatus, which at first must be put on loose, and gradually tightened; from time to time to time it is taken off, and the foot washed with camphorated spirits of wine, to prevent excoriation. Generally in four or six weeks the foot becomes straight enough to admit of a common boot being worn, with a spring to it extending up to the knee, like the one on Scarpa's machine, to which we have added another spring, which aids the bending of the foot on the instep. Besides these springs, we fasten the foot with three straps inside the boot, in its proper position. After this boot has been worn for four or six weeks, the patient generally can wear a common boot without any further precaution.

The treatment above described requires modification in different cases. The operation, for instance, is not at all limited to the division of the tendo-achillis, sometimes the tendon of the tibialis posticus muscle has to be divided, sometimes the flexor longus pollicis, and different others, if they are so contracted, that a mere extending apparatus will not suffice.

We should add here that the operation has been successfully performed on a child of one year, and on a lady of fifty-three years of age; besides a great number of cases of all ages between those two periods."

Dr. Detmold reports three successful cases. The first patient was fifteen years of age, and the deformity with him was congenital. "The inner margin of the foot was nearly bent into a right angle, and when the boy stood with both his legs together, the toes of the club-foot were behind and above the inner ankle of the right sound foot." The operation was performed in the manner just detailed. Six weeks afterwards, the boy walked "on the flat sole of a straight foot." "We have now the cast of the foot before us, taken five months after the operation, and the foot is perfect without exhibiting any other anomaly, than a slight remnant of the thick callus on the outer and upper part of the foot."

His second and third cases were also congenital, and the deformity occurred in both feet. The one of fifteen, the other of fourteen years standing. Both exhibited in every respect the same degree of deformity. "Both feet are alike; there is not the slightest remnant of a calf on either side; the heel is drawn up as far as possible about four inches above the part of the foot which touches the ground, the sole of the foot looking backwards and upwards, the tarsus has taken its place, and supports the weight of the body; consequently a callus has formed there, which is about an inch thick; a line passing over the sole, from the heel to the big toe, which in a well-shaped foot would be a straight line, describes here even more than a right angle; in consequence of which the big toe appears fully two inches longer than the little toe." The same plan of treatment was pursued, and with entire success in the first case, and the second was rapidly progressing to a favorable termination. The external wound in all these cases healed in about forty-eight hours.

Dr. Detmold, we believe, has the merit of being the first to perform, in this country, the operation of Stromeyer.

